# Impact of COVID‐19 pandemic on patients with obstructing urinary stones complicated by infection

**DOI:** 10.1002/bco2.145

**Published:** 2022-03-13

**Authors:** Haim Herzberg, Ziv Savin, Rinat Lasmanovich, Ron Marom, Reuben Ben‐David, Roy Mano, Ofer Yossepowitch, Mario Sofer

**Affiliations:** ^1^ Department of Urology, Tel‐Aviv Sourasky Medical Center Sackler School of Medicine, Tel Aviv University Tel Aviv Israel

**Keywords:** calculi, COVID‐19, infection, obstruction, ureter

## Abstract

**Objective:**

To assess the influence of COVID‐19‐imposed life changes on presentation and outcomes of patients with obstructing urinary stones complicated by infection.

**Patients and methods:**

All patients presenting with obstructing urinary stones and infection 1 year before the pandemic (March 2019 to February 2020; *n* = 66) and 1 year since its onset (March 2020 to February 2021; *n* = 45) were enrolled. Demographics, clinical presentation, laboratory panel, stone characteristics and outcomes were compared between groups. Univariate and multivariate logistic regression models were performed for analysis.

**Results:**

The COVID‐19 period was characterised by younger patients, female predominance, higher temperature at presentation and more bilateral obstructing stones (*p* < 0.05). The admission rate to intensive care units was double that of the pre‐pandemic period, whereas time between diagnosis and treatment was similar. The univariate analysis revealed higher rates of severe sepsis (odds ratio [OR] = 3, *p* = 0.01), systemic inflammatory response syndrome (SIRS) ≥ 2 (OR = 2.9, *p* = 0.01) and risk, injury, failure, loss of kidney function and end‐stage kidney (RIFLE) criteria ≥ 1 (OR = 2.2, *p* = 0.04) in the pandemic period group. The multivariate analyses revealed the COVID‐19 period as being the sole variable associated with severe sepsis (OR = 3.1, *p* = 0.02), SIRS ≥ 2 (OR = 3.8, *p* = 0.005) and RIFLE ≥ 1 (OR = 2.6, *p* = 0.05).

**Conclusions:**

The pandemic period was characterised by a worse clinical state at presentation of patients with obstructing urinary stones complicated by infection, probably reflecting delay in arrival to emergency services.

## 1 INTRODUCTION

The global COVID‐19 crisis led to enormous changes in the delivery of healthcare. The lockdowns that were imposed to control disease spread also contributed to difficulties in providing medical care. Public anxiety from exposure to the COVID‐19 virus was also contributory to delays in diagnosis and treatment for various non‐COVID‐19‐related emergencies.^1^, ^2^, ^3^, ^4^, ^5^, ^6^, ^7^ This situation required reorganisation and modification in both elective and urgent medical prioritisation. Urologists practising in countries strongly affected by the first wave of the pandemic dealt with the immediate need to reclassify the levels of urgency of all key diagnoses and treatments. They produced altered recommendations and guidelines in order to overcome the unique challenges of the pandemic's effects and to ensure effective and timely urologic care.^8^, ^9^, ^10^, ^11^

Stone disease, although benign in nature, can have detrimental effects on quality of life by causing pain and leading to disability. It may affect kidney function and carry a substantial risk of infective complications. The kinds of infections related to obstructing urinary stones represent a potentially life‐threatening medical emergency requiring urgent antibiotic treatment and kidney drainage. Delay in treatment and diagnosis has proved to be related to increased risk of mortality and morbidity.^12^, ^13^, ^14^, ^15^ As such, without exception, all recent guidelines published during the COVID‐19 pandemic defined this situation as ‘emergent’.^8^, ^9^, ^10^, ^11^

Previous studies that had evaluated the pandemic's impact on stone disease showed that there were significantly fewer patients seeking medical aid.^16^, ^17^, ^18^, ^19^ However, there is a paucity of information on the presentation for medical assistance on the part of patients with emergent stone‐related infection. This study aimed to assess the influence of the COVID‐19 pandemic on the presentation, evaluation and outcomes of patients with obstructing urinary stones complicated by infection.

## 2 PATIENTS AND METHODS

The study was approved by the institutional review board (No. 0072‐21). It comprised all 111 patients presenting to the emergency room (ER) with obstructing ureteral stones and signs of urinary tract infection between 1 March 2019 and 28 February 2021. Their medical files were retrospectively retrieved for analysis. Two comparison groups were created according to timeframes: before COVID‐19 (1 March 2019 to 29 February 2020; *n* = 66 patients) and during the COVID‐19 crisis (1 March 2020 to 28 February 2021; *n* = 45 patients). A computerised database was extended to include demographics, clinical characteristics at presentation (body temperature, blood pressure, heart rate and visual analogue scale [VAS] pain scoring scaled from 1 to 10), basic laboratory panel (blood count, creatinine, electrolytes, C‐reactive protein [CRP], urine microscopy and urine and blood cultures), stone characteristics (side, size, location and density in Hounsfield units [HU]) and subsequent clinical outcomes.

The study inclusion criteria were the presence of obstructing ureteral stones diagnosed by non‐contrast computed tomography (NCCT) and at least one of the following signs of infection: fever (≥38°C) within 24 h prior the presentation and urine microscopy with ≥500 white blood cells (WBCs) per field and/or positive nitrites. All patients presenting during the pandemic period were assessed by rapid polymerase chain reaction (PCR) tests for COVID‐19 at presentation, during hospitalisation and at release from the hospital. Once the diagnosis of a urinary infection was established, the patients underwent kidney drainage by retrograde ureteral stent insertion or, in case of failure of that procedure, by percutaneous nephrostomy.

### 2.1 Outcomes

Outcomes were analysed by infection severity, admission to the intensive care unit (ICU) and overall hospital stay. Severity of infection was measured by the systemic inflammatory reaction syndrome (SIRS) criteria (negative ≤1 vs. positive ≥2), by severe sepsis as defined by organ dysfunction, hypotension or hypoperfusion and by the presence of bacteremia and/or bacteriuria.^20^, ^21^, ^22^, ^23^ Kidney injury was measured by the risk, injury, failure, loss of kidney function and end‐stage kidney disease (RIFLE) criteria.^24^

### 2.2 Statistical analysis

Descriptive statistics were used to assess patient characteristics. Continuous variables were reported as median and interquartile range (IQR) and compared between groups by means of the Mann–Whitney *U* test. Categorical variables were reported as frequencies and compared with Fisher's exact and chi‐squared tests. Univariate and multivariate logistic analyses were performed to investigate the correlation between study outcomes and timeframe groups. All statistical analyses were two**‐**sided, and significance was defined as *p* < 0.05. SPSS software (IBM SPSS Statistics, Version 25, IBM Corp., Armonk, NY, USA, 2017) was used for all statistical analyses.

## 3 RESULTS

The study patients' baseline characteristics are summarised in Table 1. The COVID‐19 period group was characterised by younger patients (median age 63.5 vs. 71.5 years for the pre‐COVID‐19 group, *p* = 0.02), female predominance (73% vs. 50%, respectively, *p* = 0.02), higher temperature at presentation (median of 38.1°C vs. 37.6°C, *p* = 0.04) and more bilateral obstructing stones (18% vs. 9%, *p* = 0.04). In addition, there was a trend towards increased pain and heart rate, however, not statistically significant (*p* = 0.08 for both). No differences were found in baseline kidney function as measured by the calculated estimated glomerular filtration rate (eGFR), WBC counts, CRP values and stone characteristics. The time that had elapsed from presentation to NCCT and kidney drainage did not differ significantly between the study groups (*p* = 0.68 and *p* = 0.08, respectively). Retrograde drainage by an internal stent was performed in 110 patients, and it failed in one patient who then underwent percutaneous nephrostomy.

**TABLE 1:** Demographic, clinical and laboratory characteristics at presentation

	Pre‐COVID‐19 group (*n* = 66)	COVID‐19 group (*n* = 45)	*p* value
Age, years; median (IQR)	71.5 (51.5–80)	63.5 (49.2–73.2)	**0.02**
Sex (male, %)	33 (50%)	12 (27%)	**0.02**
Diabetes mellitus, *n* (%)	21 (31%)	15 (33%)	0.51
History of nephrolithiasis, *n*	30 (45%)	17 (38%)	0.44
History of stones treatment, *n*	9 (14%)	11 (24%)	0.21
eGFR at presentation, ml/min; median (IQR)	53 (39–72)	41 (31–69)	0.14
Laterality, *n*			**0.04**
Right	39 (49%)	16 (36%)	
Left	21 (32%)	21 (46%)	
Bilateral	6 (9%)	8 (18%)	
Stone size, mm; median (IQR)	7.0 (5–9.7)	6.4 (5.1–10)	0.38
Stone density, Hounsfield units; median (IQR)	745 (452–987)	740 (488–1153)	0.55
Pain (VAS)			0.08
0–3	33	18	
4–10	23	24	
Temperature, °C; median (IQR)	37.4 (36.7–38.7)	38.1 (37.4–38.8)	**0.04**
Heart rate, pulse/minute; median (IQR)	95 (84–110)	103 (84.2–116)	0.08
WBC, 10^3^/ml; median (IQR)	12.95 (9.8–17.5)	12.4 (9.8–18.6)	0.75
CRP, mg/L; median (IQR)	90 (33.7–142)	97.5 (24.6–190)	0.36
Time to NCCT, hours; median (IQR)	6 (3–16.2)	5 (3–10)	0.68
Time to drainage, hours; median (IQR)	15 (10–47.5)	11 (7–20.5)	0.08
ICU hospitalisation	7 (11%)	10 (22%)	0.11

*Note*: Bold indicates significant.

Abbreviations: °C, degree Celsius; CRP, C‐reactive protein; eGFR, estimated glomerular filtration rate (mL/min/1.73 m^2^); ICU, intensive care unit; IQR, interquartile range; NCCT, non‐contrast computerised tomography; VAS, visual analogue scale; WBC, white blood cell count.

The univariate logistic regression analyses revealed that the COVID‐19 period was associated with higher rates of severe sepsis (OR = 3, 95% confidence interval [CI] 1.3–6.7, *p* = 0.01), SIRS ≥ 2 (OR = 2.9, 95% CI 1.3–6.6, *p* = 0.01) and RIFLE ≥ 1 (OR = 2.2, 95% CI 1–4.9, *p* = 0.04). There was a significant association between eGFR and severe sepsis, as well as age and RIFLE ≥ 1. The multivariate analyses revealed that the COVID‐19 era remained the sole clinical variable associated with severe sepsis (OR = 3.1, 95% CI 1.2–8.2, *p* = 0.02), SIRS ≥ 2 (OR = 3.8, 95% CI 1.5–9.8, *p* = 0.005) and RIFLE ≥ 1 (OR = 2.6, 95% CI 1–7.2, *p* = 0.05) (Table 2).

**TABLE 2:** Univariate and multivariate logistic regression models for severe sepsis, SIRS and RIFLE criteria

Variables	Univariate analysis			Multivariate analysis		
	OR	95% CI	*p*	OR	95% CI	*p*
Severe sepsis						
Age	1	0.99–1	0.12	1	0.9–1	0.13
Sex	1.31	0.6–2.9	0.51	1.27	0.5–3.2	0.61
eGFR at presentation	**0.98**	**0.9–1**	**0.04**	0.99	0.9–1	0.36
Laterality	1.43	0.8–2.5	0.21	1.13	0.6–2.1	0.70
COVID‐19 era	**2.97**	**1.3–6.7**	**0.01**	**3.15**	**1.2–8.2**	0.02
SIRS ≥ 2						
Age	1	0.9–1	0.88	1	0.9–1	0.22
Sex	1.43	0.6–3.1	0.36	1.12	0.5–2.6	0.80
eGFR at presentation	1	0.9–1	0.40	1	0.9–1	0.15
Laterality	1.1	0.6–1.8	0.78	0.93	0.5–1.7	0.82
COVID‐19 era	**2.92**	**1.3–6.6**	**0.01**	**3.85**	**1.5–9.8**	0.005
RIFLE ≥ 1						
Age	**1.03**	**1–1.1**	**0.008**	1	0.9–1	0.21
Sex	0.61	0.3–1.3	0.21	0.61	0.2–1.5	0.31
eGFR at presentation	**0.96**	**0.9–1**	**<0.001**	**0.97**	**0.9–1**	0.006
Laterality	**1.9**	**1–3.4**	**0.02**	1.3	0.6–2.5	0.43
COVID‐19 era	**2.24**	**1–4.9**	**0.04**	**2.64**	**1–7.2**	0.05

*Note*: Bold indicates significant.

Abbreviations: CI, confidence interval; eGFR, estimated glomerular filtration rate (mL/min/1.73 m^2^); OR, odds ratio; RIFLE, risk, injury, failure, loss of kidney function and end‐stage kidney disease; SIRS, systemic inflammatory reaction syndrome.

The rates of bacteremia, bacteriuria and septic shock were similar for both study groups. The ICU admission rate during the pandemic was double that of the previous period; however, this trend did not reach a level of statistical significance (22% during COVID‐19 vs. 11% before COVID‐19, *p* = 0.11). The average hospital stay was 7 days for both groups (*p* = 0.9). None of the patients in the pandemic era group was COVID‐19‐positive at presentation nor had become positive during hospitalisation.

## 4 DISCUSSION

The COVID‐19 pandemic has been characterised by an exponential increase in the need for hospitalisation, a considerable burden on ICUs for advanced resuscitative interventions, assisted ventilation or use of extracorporeal membrane oxygenation (ECMO) and an expanded number of beds needed for rehabilitation programmes.^25^ These sudden changes resulted in quick adaptive measures taken by medical systems, including modifications to the ER triage process, reduction or cancellation of elective medical activities, transfer of medical personnel from other medical fields to the newly organised designated COVID‐19 spaces and re‐directing much of the medical budgetary resources to fund these alterations. In addition, general national steps, such as lockdowns and quarantining, as well as alarming mass media reports—possibly exaggerated on occasion—induced an atmosphere of panic that resulted in restraints and delays in the public's seeking of medical aid for non‐pandemic‐related morbidities.

One U.S. study reported that 40.9% of adults having avoided medical care during the pandemic because of concerns about exposure to COVID‐19, including 12.0% who avoided urgent or emergency care and 31.5% who avoided routine care.^4^ A multicentre study that assessed the impact of the pandemic on patients with ST‐elevation myocardial infarction revealed a 19% reduction in primary percutaneous coronary interventions, a longer delay to treatment and a 20% increase in related mortality.^1^ A group from Germany reported a fourfold increase in mortality related to myocardial infarction in the pandemic period.^5^ Similar findings were observed in patients with stroke, with up to a 30% decrease in presentation and interventions and fewer patients presenting with mild stroke.^7^ Likewise, over 60% of newly diagnosed patients with type 1 diabetes presented with diabetic ketoacidosis during the pandemic.^6^ In oncology, Patt et al. demonstrated that screening for cancer was reduced by up to 50% and that cancer patient intake and follow‐up decreased by up to 40%.^2^ Modelling these findings on English patients resulted in a predicted additional cancer mortality of 5–17%.^3^

In urology, we witnessed efforts to adapt to the rapidly changing medical reality with the creation of guidelines for addressing clinical urologic scenarios and processes of clinical prioritisation. Goldman and Haber^8^ and Proietti et al.^9^ published clearcut clinical criteria for endourology and emergent urological surgery, respectively. Both reports defined drainage of infected and obstructed kidneys as a medical emergency that must be treated immediately, even during the pandemic. This recommendation was supported by studies that had been published before the pandemic, showing that delayed decompression is associated with higher rates of morbidity and mortality.^12^, ^13^, ^14^, ^15^ Several studies assessed the COVID‐19 effect on kidney stone disease. Kachroo et al. showed a 36% decrease in emergent stone disease presentation during the COVID‐19 pandemic, accompanied by higher rates of acute kidney injury.^16^ Some of these findings were supported also by Gul et al. who showed higher rates of kidney injury, high grades hydronephrosis and cases classified as emergent by guidelines issued during the pandemic.^26^ In a review summarising current worldwide urologic trends to help urologists in decision making during the COVID‐19 pandemic, Tonyali et al. outlined the urgency of draining obstructed kidneys by placing either stents or nephrostomy tubes. Those authors noted that the lack of mechanical ventilators because of their being used for COVID‐19 patients may necessitate that these procedures be performed under local anaesthesia.^17^

During the pandemic, patients with stone events reported delay in seeking medical aid and in arriving to the ER. Those with obstructing pyelonephritis may have had higher complications rates and SIRS levels, and such delays were translated into higher rates of morbidity and mortality.^18^, ^19^ A plausible explanation for the patients' reluctance to seek emergency care appears to be public anxiety about exposure to the virus. This issue was not addressed in depth by medical organisations until late into the pandemic, when instructive medical messages started to emerge through mass media.

We believe that our comparative study that followed a year‐long influence of pandemic on characteristics and outcome of patients presenting with stone obstruction complicated by infection provides additional and important insights to the sparse literature on these issues. Although we did not directly address the issue of delay in presentation, our patients who presented to the ER during the pandemic had a more severe clinical and biochemical grade of infection, suggesting that they had refrained from seeking treatment. The multivariate analysis revealed the COVID‐19 period as the single factor determining an around threefold greater severity of sepsis, SIRS and RIFLE in comparison with the period before the pandemic. On the other hand, the treatment was provided in a timely manner and with successful outcome, attesting to good institutional organisation despite the COVID‐19 impact on our healthcare system. Although the difference in our ICU admission rates did not reach a level of significance despite the twice higher rate during the pandemic (22% vs. 11%), it should be kept in mind that the greater burden on the ICUs probably determined a stricter triage of patients, resulting in the deferring of some of them to other medical facilities. Unlike our experience, however, other groups providing proper therapy timelines reported inferior clinical outcome, again suggesting a negative impact of patient delay in presentation.^12^, ^13^, ^14^, ^15^, ^19^

We are aware that our study has some limitations. First, it represents the experience of a single tertiary referral medical centre in a highly developed urban region with sufficient resources to cope with regular emergencies as well as the extra burden caused by COVID‐19‐infected patients. It is possible that the outcomes would have been different in lesser endowed circumstances. However, our experience showed that shifting resources to ensure medical care for pandemic needs and still provide urgent interventions should begin with reducing elective procedures.^27^, ^28^ Second, the retrospective design of this study can pose a limitation although we believe that the sudden onset, the unpredictable course, and the confusion among the pandemic prediction models precluded any possibility to initiate a prospective comparison of medical issues related to COVID‐19.

## 5 CONCLUSIONS

The first year of the COVID‐19 pandemic was characterised by patient delay in seeking emergency care for infections related to obstructive urinary stones as well as by worse clinical states at presentation. Although well‐coordinated and optimised resources were able to maintain the capabilities to accommodate these cases with good results, the public will need to be convinced that treatment for other emergencies can be delivered safely without increased risk of COVID‐19 exposure.

## CONFLICT OF INTEREST

No competing financial interests exist.

## AUTHOR CONTRIBUTIONS

Haim Herzberg was responsible for the design, data acquisition and drafting. Ziv Savin did the statistical assessment. Rinat Lasmanovich performed the data acquisition. Ron Marom did the data acquisition and computerization of data. Reuben Ben‐David performed the data acquisition and completion of ethical requirements. Roy Mano did the scientific supervision. Ofer Yossepowitch was responsible for the scientific and ethical supervision. Mario Sofer did the overall supervision and final editing.
